# Allopregnanolone preferentially induces energy‐rich food intake in male Wistar rats

**DOI:** 10.14814/phy2.12190

**Published:** 2014-12-11

**Authors:** Ellinor Holmberg, Maja Johansson, Torbjörn Bäckström, David Haage

**Affiliations:** 1 Department of Clinical Sciences, Obstetrics and Gynaecology, Umeå Neurosteroid Research Center, Umeå University, Umeå, Sweden

**Keywords:** Allopregnanolone, energy need, food intake, neurosteroids GABA

## Abstract

Obesity is an increasing problem and identification of the driving forces for overeating of energy‐rich food is important. Previous studies show that the stress and sex steroid allopregnanolone has a hyperphagic effect on both bland food and palatable food. If allopregnanolone induces a preference for more palatable or for more energy‐rich food is not known. The aim of this study was to elucidate the influence of allopregnanolone on food preference. Male Wistar rats were subjected to two different food preference tests: a choice between standard chow and cookies (which have a higher energy content and also are more palatable than chow), and a choice between a low caloric sucrose solution and standard chow (which has a higher energy content and is less palatable than sucrose). Food intake was measured for 1 h after acute subcutaneous injections of allopregnanolone. In the choice between cookies and chow allopregnanolone significantly increased only the intake of cookies. When the standard chow was the item present with the highest caloric load, the chow intake was increased and allopregnanolone had no effect on intake of the 10% sucrose solution. The increased energy intakes induced by the high allopregnanolone dose compared to vehicle were very similar in the two tests, 120% increase for cookies and 150% increase for chow. It appears that in allopregnanolone‐induced hyperphagia, rats choose the food with the highest energy content regardless of its palatability.

## Abstract (precis)

We show that allopregnanolone increased the consumption of the more calorie dense food offered in different food preference situations, while palatability was of secondary importance. Thus, it seems as allopregnanolone treatment could favor ingestion of a more energy‐rich diet.

## Introduction

More and more people are struggling with being overweight and obese; hence, understanding the underlying mechanisms of our eating behavior is of great importance. Eating is vital to every living animal and is, therefore, under strong regulatory control. Although eating less food than energy requirements is hard to obtain, the opposite, excessive eating is rather easy and it has been suggested that obese subjects have a weaker input from satiety signals (Lawton et al. 1993). However, bland food is rarely eaten in excess, which contrasts to palatable food that is often eaten beyond energy requirements (Kenny 2011). Therefore, food intake can be determined by other factors than pure energy requirements. Indeed, today the general idea is that food intake is driven both by the reward a palatable food can give and the need for energy (Goldstone et al. 2009; Berthoud 2011).

Our group and others have previously shown that the neurosteroid allopregnanolone has a hyperphagic effect on both bland foods, as well as, on palatable food (Chen et al. 1996; Reddy and Kulkarni 1998, 1999; Holmberg et al. 2013). However, in these studies, food items were presented singly and it is not possible to evaluate if allopregnanolone induced a preference for certain types of food. In this study, we therefore address the questions; Does allopregnanolone make any difference in the rat's food preference? Does allopregnanolone especially increase ingestion of more palatable food or of food with higher energy content?

To elucidate the above questions, we adapted a protocol that has previously been used to investigate peptide‐induced food choice between energy dilute yet more palatable food items and energy rich but less palatable food items. In those studies certain peptides associated with food intake regulation increased the consumption of palatable calorie dilute food, a 10% sucrose solution, while other peptides instead increase the consumption of bland calorie dense food, standard chow (Giraudo et al. 1999; Olszewski et al. 2003; Bomberg et al. 2007). The caloric density of the sucrose solution, 0.4 kcal·g^−1^, is similar to that in most available sugar‐sweetened beverages, and is less satiating than sugar in solid form (DiMeglio and Mattes 2000). In humans, allopregnanolone has previously been associated with binge eating attacks, a condition usually associated with consumption of highly palatable and energy dense food items (Hadigan et al. 1989; Guertin and Conger 1999; Monteleone et al. 2003). For rats one such highly preferred food is chocolate chip cookies (Rothwell and Stock 1979; Rolls et al. 1980).

Allopregnanolone is present in both sexes and elevated plasma levels of allopregnanolone have been detected in both obese men and obese women compared to normal weight controls (Menozzi et al. 2002). Allopregnanolone is a stress steroid and increases both in serum and brain tissue during stress (Purdy et al. 1991; Droogleever Fortuyn et al. 2004). Since allopregnanolone is a progesterone metabolite, the level of allopregnanolone in women is increased in the luteal phase of the menstrual cycle, a phase when also food intake is elevated and binge eating is more frequent (Johnson et al. 1994; Barr et al. 1995; Nyberg et al. 2007; Klump et al. 2008).

Allopregnanolone is a neurosteroid with specific binding sites at the gamma‐aminobutyric acid (GABA) _A_‐receptor where it acts as a potent positive modulator of the receptor (Majewska et al. 1986; Morrow et al. 1990; Paul and Purdy 1992; Lambert et al. 1995, 2003; Belelli et al. 2006; Hosie et al. 2007). GABA has recently been identified as an important transmitter of Agouti‐related peptide (AgRP) expressing neurons which are considered integrators of various nutritional, hormonal, and neuronal signals involved in food intake. In fact, GABA‐ergic transmission from AgRP neurons has been suggested to have an important role to ensure sufficient food intake and weight gain (Morton et al. 2006; Tong et al. 2008; Wu et al. 2009). Furthermore, GABA‐ergic transmission from AgRP neurons onto downstream paraventricular (PVN) neurons appears to be especially important for acute AgRP‐neuron‐evoked food intake (Atasoy et al. 2012). Therefore, it can be speculated that allopregnanolone acts at postsynaptic GABA_A_ receptors to influence food intake. Benzodiazepines, that similar to allopregnanolone act as positive GABA_A_ receptor modulators, induce increased intake preferentially of palatable foods (Berridge and Pecina 1995; Cooper 2005). In line with these findings, compounds that have the opposite effect, acting as GABA_A_ receptor inverse agonists reduced intake of palatable food (Cottone et al. 2007). Since benzodiazepines and allopregnanolone share many properties of action at the GABA_A_ receptor, allopregnanolone might induce a similar increase in palatable food intake. On the other hand, in previous reports about GABA‐ergic action on food intake, GABA has been associated with an increase of overall energy intake (Basso and Kelley 1999; Echo et al. 2002; Khaimova et al. 2004; Woolley et al. 2006). Thus, it is still an open question if allopregnanolone mainly will induce increased intake of energy dense, palatable food, or both. In this study, we try to elucidate this question.

## Materials and Methods

### Ethical approval

All experimental procedures, treatments, and housing were conducted in accordance with national guidelines for use of laboratory animals. The experimental protocols were approved by the Ethical Committees for Animal Studies, Umeå, Sweden.

### Animals

Forty‐eight adult male Wistar rats (Taconic, Ry, Denmark) were group‐housed in 55 cm × 35 cm × 20 cm plastic cages, three rats per cage, under a 12‐h dark–light cycle with lights off at 10.00 h and lights on at 22.00 h. Standard chow and water were available ad libitum. Each animal was identified by a mark made on its tail with a permanent marker. To avoid putative reported antineophobic effects induced by allopregnanolone (Fudge et al. 2006), care was taken to acclimatize the rats to the environment and to different diets. Two weeks prior to the first experiment, the rats had, in addition to standard chow and water, ad libitum access to a 10% sucrose solution or to Maryland Original^™^ chocolate chip cookies in their home cages. Maryland Original^™^ chocolate chip cookies were used as they are well known internationally. The intake of sucrose solution and cookies were stable prior to the onset of the experiments. To minimize stress, all rats were repeatedly handled and habituated to new environments and procedures to be applied in the experiments. The habituation included spending 1 h alone in a separate smaller test cage, 40 × 25 × 15 cm at repeated occasions with ad libitum access to the different food types and water. Male rats were chosen to avoid the endogenous allopregnanolone fluctuations from the female estrous cycle and the estrous cycle‐dependent effect of allopregnanolone on food intake (Reddy and Kulkarni 1999; Frye et al. 2000).

### Solutions

The vehicle used was 10% 2‐hydroxypropyl‐*β*‐cyclodextrine (*β*‐cd) (Sigma Chemical Co., St. Louis, MO). Allopregnanolone (3*α*‐hydroxy‐5*α*‐pregnan‐20‐one) was obtained from Umecrine AB (Umeå, Sweden) and dissolved in 10% *β*‐cd by ultrasonication. NaCl was added to make the solutions isotonic. As detailed below, two doses were applied in the experiments (1.25 and 2.5 mg·kg^−1^ body weight), and to keep the injection volume constant and low (1 mL·kg^−1^ body weight), solutions with different allopregnanolone concentrations were used for the different doses. The levels of allopregnanolone generated by exogenous administration at these doses are reported in detail in our previous study (Holmberg et al. 2013).

### Experimental design

All tests were performed in the same room where the rats were kept. Two separate experiments were performed. In both experiments, food intake was measured for 1 h. Rats then had access to the two food types offered in that experiment. The experimental design was similar to that used in other studies to discriminate between palatable food and energy‐rich food (Giraudo et al. 1999; Olszewski et al. 2003; Bomberg et al. 2007). In the choice between standard chow and a 10% sucrose solution, the rats (*n* = 24) weighed 394 ± 27 g at the onset of the experiments. In the other experiment, the rats (*n* = 24) weighed 375 ± 14 g at the onset of the experiments and they were provided standard chow and Maryland Original^™^ chocolate chip cookies.

Food intake was measured individually in single cages. There was a wash‐out period of at least 3 days between trials. The rats were allocated into three groups, eight animals per group. At each test occasion, one group received s.c. injection of vehicle, while the other two groups received allopregnanolone at one of the two doses (1.25 or 2.5 mg·kg^−1^ body weight). We used a cross‐over design and the procedure was repeated three times (on different days) so that every animal received all treatments. All experiments were performed at the onset of the rats' dark/active period. At this time point, the rats' rates of food intake are highest, and administration of allopregnanolone has strongest effects concerning eating (Farley et al. 2003; Holmberg et al. 2013). Following injections, the rats were placed alone in the test cages. After 10 min, each rat was allowed 1 h free access to standard chow, a bottle with 10% sucrose solution, and a water bottle, or to standard chow, cookies, and a water bottle. The rats were then transported back to their home cages and each cage used in the experiment was thoroughly searched for possible food spillage. The amount of food consumed was weighed and converted into kcal using the following approximations: standard chow, 3.5 kcal·g^−1^; sucrose solution, 0.4 kcal·g^−1^; and cookies, 5.2 kcal·g^−1^. The fat content of the standard chow was less than 5%, while the cookies contained 25% fat. The rats' water intake was also measured.

### Statistical analysis

For statistical analysis, all measures of consumed amounts (weights) of the offered foods were converted into kcal. Each experiment was repeated three times with cross‐over of the three treatments (*n* = 8 per treatment at each replicate), and the food consumption data are presented as means ± SEM (*n* = 24 per treatment for each food choice). Data were analyzed using SPSS version 18 (SPSS Inc., Chicago, IL) and Graph Pad Prism (Graph Pad Software, Inc. La Jolla, CA) software.

Nonparametric statistics was used since some of the data showed skewed distributions. Friedman's test, which is similar to one‐way analysis of variance (ANOVA) with repeated measures, was used to compare the intake of the offered foods and solutions between the different treatments. Friedman's test was followed post hoc with paired sample Wilcoxon signed‐rank test, adjusted by Bonferroni correction.

To detect differences between total energy intake of the different food types over the different dosages in the cookie/chow experiment, the area under the curve (AUC) for intake of each food type was calculated (kcal × treatment) for each animal and compared by the paired sample Wilcoxon signed‐rank test.

Mann–Whitney *U*‐tests, adjusted by Bonferroni correction, were used to compare total liquid intake for the vehicle‐treated animals and total kcal intake between the two different experiments. The total kcal intake in the two experiments, as well as the increase in food intake by allopregnanolone injections, was also compared to a previous study where chow was given as the sole food source (Holmberg et al. 2013) using this test.

## Results

### Allopregnanolone increased consumption of standard chow rather than the intake of the less caloric and more palatable sucrose solution

**Figure 1.**
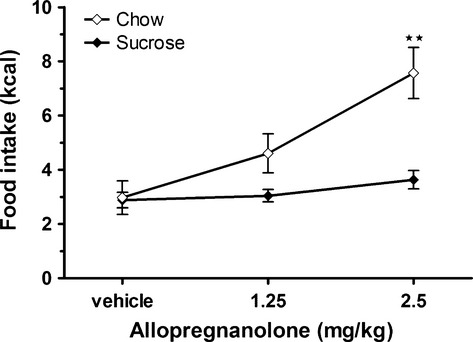
**Allopregnanolone significantly increased the energy intake from standard chow but not from 10% sucrose solution in a food choice test.** Consumption of standard chow and 10% sucrose solution were measured for 1 h after subcutaneous injections of allopregnanolone (1.25 or 2.5 mg·kg^−1^ body weight) or vehicle. Data is presented as means ± SEM (*n* = 24 per treatment). Significant differences compared to the corresponding vehicle are shown as **(*P* ≤ 0.01).

When the rats had access to both standard chow, which is relatively energy‐rich but with a bland taste, and a 10% sucrose solution, which is energy dilute but more palatable, allopregnanolone injections significantly increased chow intake (chi‐sq (df = 2) = 15.12; *P* ≤ 0.001). 2.5 mg·kg^−1^ allopregnanolone increased the energy intake by 4.6 kcal compared to vehicle (*Z* = −3.23; *P* ≤ 0.01; Fig. 1). The chow intakes after the different treatments were as follows: vehicle 3.0 ± 0.6 kcal, 1.25 allopregnanolone 4.6 ± 0.7 kcal, and 2.5 allopregnanolone 7.6 ± 0.9 kcal. When treated with allopregnanolone 2.5 mg·kg^−1^, the rats ate 155% more from chow compared to the intake from chow after vehicle treatment. When compared to the total kcal intake (both chow and sucrose) after vehicle treatment, the increase was 78%. No significant difference in intake of sucrose solution was detected among the three treatments (vehicle 2.9 ± 0.3 kcal, 1.25 allopregnanolone 3.0 ± 0.2 kcal, and 2.5 allopregnanolone 3.6 ± 0.3 kcal).

### Allopregnanolone increased consumption of calorie dense and more palatable cookies rather than the consumption of standard chow

**Figure 2.**
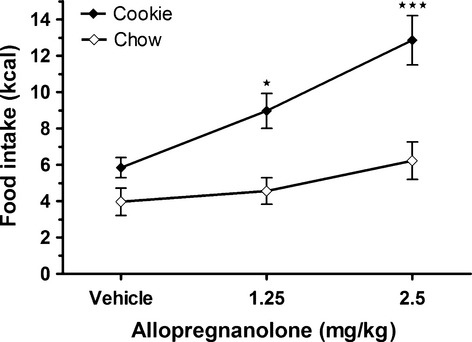
**Allopregnanolone significantly increased the energy intake from chocolate chip cookies but not from standard chow in a food choice test.** Consumption of chocolate chip cookies and standard chow was measured for 1 h after subcutaneous injections of allopregnanolone (1.25 or 2.5 mg kg^−1^ body weight) or vehicle. Data is presented as means ± SEM (*n* = 24 per treatment). Significant differences compared to the corresponding vehicle are shown as *(*P* ≤ 0.05) and ***(*P* ≤ 0.001).

When rats were provided chocolate chip cookies and standard chow allopregnanolone injections, overall significantly altered the intake of cookies (chi‐sq (df = 2) = 18.07; *P* ≤ 0.001), but not the intake of chow. The cookie consumption after the different treatments was as follows: vehicle 5.8 ± 0.6 kcal, 1.25 allopregnanolone 9.0 ± 1.0 kcal, and 2.5 allopregnanolone 12.9 ± 1.4 kcal, while the intake of chow was as follows: vehicle 4.0 ± 0.7 kcal, 1.25 allopregnanolone 4.6 ± 0.7 kcal, and 2.5 allopregnanolone 6.2 ± 1.0 kcal. Furthermore, the increase in cookie intake was dose dependent (Fig. 2), equivalent to 3.1 and 7.0 kcal per rat, respectively, following allopregnanolone 1.25 mg·kg^−1^ (*Z* = −2.52; *P* ≤ 0.05) and 2.5 mg·kg^−1^ body weight (*Z* = −3.58; *P* ≤ 0.001), injections. The increased cookie intake by 2.5 mg·kg^−1^ allopregnanolone yielded an increase of 120% when compared to the cookie intake after vehicle treatment. When compared to the total kcal intake (both cookie and chow) after vehicle treatment the increase was 71%.

To compare the total energy intake over dosages from cookies with the total energy intake from chow, the area under the curve (AUC, dose × intake) was studied. This was done since the energy consumption in the vehicle situation differed between the standard chow and cookies. A comparison of the AUC between cookie intake and chow intake for all treatments showed that overall, the rats had significantly higher kcal intakes from cookies than chow (*Z* = −3.17; *P* ≤ 0.01). The intake of cookies was larger than the chow intake both in kcal and in weight (gram) (data not shown).

### The consumption of sucrose solution was not simply a substitute for water

**Figure 3.**
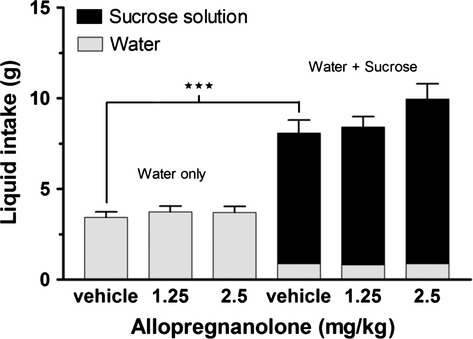
**Allopregnanolone did not significantly alter the water intake.** In addition to food intake (Figs. 1 and 2) the consumption of liquid was also measured in both experiments for 1 h after subcutaneous injections of allopregnanolone (1.25 or 2.5 mg·kg^−1^) or vehicle. Data is presented as means ± SEM (*n* = 24 per treatment). Water intake was not significantly affected by allopregnanolone but the animals consumed higher total volumes of liquids when they had access to both water and sucrose solutions in the chow/sucrose experiment compared to in the chow/cookie experiment when water was the sole liquid source. Significant difference is shown as ***(*P* ≤ 0.001).

Total liquid intake in the two experiments was investigated to determine if the rats when sucrose was present simply replaced their water intake with intake of sucrose solution. The rats consumed significantly more liquid when they had access to both water and sucrose solutions (vehicle 8.1 ± 0.7 g, 1.25 allopregnanolone 8.4 ± 0.6 g, and 2.5 allopregnanolone 10.0 ± 0.8 g), compared to when water was the sole liquid source (vehicle 3.4 ± 0.3 g, 1.25 allopregnanolone 3.7 ± 0.3 g, and 2.5 allopregnanolone 3.7 ± 0.3 g; *Z* = −4.98; *P* ≤ 0.001, consumption after the vehicle injections; Fig. 3). With sucrose present the rats consumed less water but the intake of sucrose solution was greater than the intake of water in experiment 2, and the water intake accounted for approximately 10% of the total liquid intake. The water intake was not significantly altered by allopregnanolone in either experiment.

### The allopregnanolone‐induced increased energy intake of the preferred food item was of the same magnitude in both choice situations

**Figure 4.**
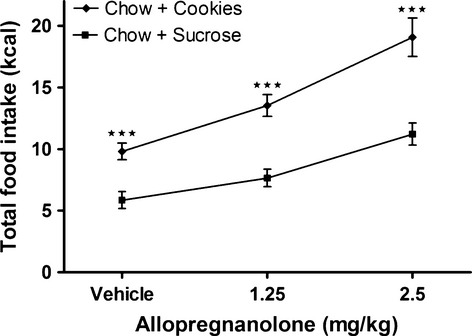
**Total calorie intake was significantly higher in the chow and chocolate chip cookies food choice test.** Total intake of calories was measured for 1 h after subcutaneous injections of either allopregnanolone (1.25 or 2.5 mg·kg^−1^) or vehicle in the two food choice tests, as indicated. Data is presented as means ± SEM (*n* = 24 per treatment). Significant differences comparing total mean intake when chow and cookies were the food sources compared to when chow and sucrose were the food sources, separately for each treatment, are shown as ***(*P* ≤ 0.001).

For all treatments, the total calorie intake was higher when chow and cookies were present (vehicle 9.8 ± 0.7 kcal, 1.25 allopregnanolone 13.5 ± 0.9 kcal, and 2.5 allopregnanolone 19.1 ± 1.6 kcal), compared to when chow and sucrose were present (Fig. 4; vehicle (5.9 ± 0.7 kcal, *Z* = −3.71; *P* ≤ 0.001); 1.25 mg·kg^−1^ allopregnanolone (7.6 ± 0.7 kcal, *Z* = −4.43; *P* ≤ 0.001); 2.5 mg·kg^−1^ allopregnanolone (11.2 ± 0.9 kcal, *Z* = −4.04; *P* ≤ 0.001). The total kcal intake with chow and sucrose did not differ from intakes recorded in our previous study when standard chow was given as the sole food choice (Holmberg et al. 2013). However, there was no significant difference between the increased energy intake from chow by 2.5 mg·kg^−1^ allopregnanolone when chow and sucrose was present (155% increase compared to vehicle) and the increased energy intake from cookies by 2.5 mg·kg^−1^ allopregnanolone when chow and cookies were present (120% increase compared to vehicle).

## Discussion

To our knowledge, this provides the first report of the effect of allopregnanolone in food choice situations. With a choice between cookies and chow, allopregnanolone‐treated rats increased their intake of the more energy rich and also more palatable cookies, and did not increase the intake of chow. When chow instead was the food present with the highest energy content, allopregnanolone‐treated rats increased their intake of chow and not that of the palatable low‐energy sucrose solution present. However, in the choice situation where the preferred item was also palatable, that is, cookies, the increased eating induced by allopregnanolone was not larger than when the preferred food was less palatable, that is, chow. Thus, it seems as allopregnanolone treatment increases the consumption of the most energy‐rich food available, while palatability is of secondary importance.

In the experiment where rats had a choice between chow and Maryland Original^™^ chocolate chip cookies, allopregnanolone increased the consumption of cookies, but there was no increase in chow consumed. Previously, we showed that allopregnanolone dose dependently increased the intake of chow, when chow was the only food available (Holmberg et al. 2013). However, in this study with both chow and cookies present, we saw a clear preference in that only the intake of the more energy dense as well as palatable cookies was increased. In the other experiment, the choice was then between a palatable low calorie liquid sucrose solution and solid chow with a higher energy content. In this case, allopregnanolone did not increase the consumption of the more palatable choice, but allopregnanolone instead induced a dose‐dependent and selective increase in chow consumption. Thus, also in this choice situation, allopregnanolone increased the consumption of the more energy‐rich food present, in this case the chow that is less palatable. Also, the consumption of standard chow in this experiment was increased by allopregnanolone to the same magnitude as we have observed in previous experiments with standard chow given solitary (Holmberg et al. 2013). In that study, after the first hour, allopregnanolone‐treated animals continued to consume at least as much standard chow as vehicle‐treated animals during the subsequent 3 h. Thus, there seems to be no immediate rebound effect of chow intake after allopregnanolone injections.

It could be argued that the large difference in energy content between the two energy sources in the sucrose/chow experiment (0.4 kcal·g^−1^ for sucrose and 3.5 kcal·g^−1^ for chow) would mask increased sucrose consumption due to a ceiling effect. However, in this study, we did not see any significant effect at all on sucrose consumption regardless of whether the weight or the energy content was used for calculation. Finally, the maximum amount of liquid consumed was well below the levels that have been reported in other investigations (Giraudo et al. 1999; Kendig et al. 2013). It could also be speculated that the rats had consumed so much sucrose so that they were at the limit to develop nausea and because of this did not consume any additional sucrose in response to allopregnanolone. However, several pieces of evidence make this less likely. Firstly, the chow intake was very similar to our previous experiments with no sucrose solution present (Holmberg et al. 2013). This was the case both during vehicle conditions and during allopregnanolone treatment. If the rats were suffering from nausea, one might expect the chow intake to be affected. Also, rats consume most of their food during their active/dark period, while they consume considerable less during the inactive/light period (Farley et al. 2003; Bassil et al. 2007; Holmberg et al. 2013). As we performed our experiments at the onset of the active/dark period, we knew that the rats had been eating less during the preceding period of time (the inactive/light period).

Both cookies and sucrose seem to be palatable for the rats. At baseline vehicle treatment rats choose to ingest approximately 60% of their energy from the cookies and for all treatments the total calorie intake was higher in the cookie experiment (Fig. 4). This is in accordance with previous data showing that laboratory rats increase their calorie intake when exposed to energy‐rich food (Martire et al. 2013). That cookies are palatable for rats has also been shown by others (Rothwell and Stock 1979; Rolls et al. 1980). The sucrose solution was shown to be more palatable compared to water because there was an increase in liquid intake also after vehicle treatment, compared to the situation when only water was present. The increase in liquid intake was mainly due to increased consumption of the sucrose solution. However, even though there was a larger part of the liquid intake that was from the sucrose solution, there was no increase after allopregnanolone treatment.

The main interpretation of these results is that allopregnanolone dose dependently induced a preference for more energy‐rich food, but as cookies are both the most energy rich, and also the more palatable food item, there might also be a possible reward effect involved. However, the increased energy intake from cookies induced by allopregnanolone, 71% increase, compared with total intake after vehicle treatment, is on a comparable level to the increased energy intake from chow in the chow/sucrose choice, 78% increase, compared with total intake at vehicle treatment. If a strong reward component would have been present, one might presume that the increase would have been considerable larger when the preferred food item was more palatable. Notably is also that allopregnanolone more or less exclusively increased intake of cookies, and did not have any effect on sucrose intake. It could then be speculated that the effect was mediated by an increased energy drive although a reward component cannot be completely ruled out. Rats seem to have the ability to sense the caloric value of food (Beeler et al. 2012). Also, it seems that hungry rats chose the more energy dense food compared to ad libitum‐fed rats which tend to choose the more rewarding food (Scheggi et al. 2013). Since the rats in our study were not food deprived, one could speculate that they would indeed have a preference for the more rewarding sucrose solution. Still, in this study, allopregnanolone did not increase the intake of the palatable sucrose.

Both experiments thus indicate that allopregnanolone‐treated rats will increase the consumption of the most energy‐rich food available. Thus, allopregnanolone‐treated rats increased their intake of a type of diet, like the cookies, which has been associated with both weight gain and obesity (Rolls et al. 1980). Feeding energy‐rich and palatable food has been one approach used to study the development of obesity in laboratory rodents (Rothwell and Stock 1982a,b; Mela 1996). If the results in the experiment with cookies are translated to a human situation, allopregnanolone would increase the consumption of energy‐rich food. This can have negative consequences for the individual since overeating energy rich, palatable food can induce diabetes and obesity (Ford et al. 1997; Salas‐Salvado et al. 2011). Therefore, it would be interesting, in future studies, to investigate the long‐term effects of allopregnanolone on both intake of energy‐rich food and weight gain.

Several sex steroids have been discussed in association with food intake (Asarian and Geary 2006). Allopregnanolone is a metabolite to one of the sex steroids, progesterone, and in women it fluctuates regularly during the menstrual cycle. The concentration of allopregnanolone peaks during the luteal phase of menstrual cycle (Nyberg et al. 2007) and it has also been reported that the food intake and frequency of binge eating attacks increase in the luteal phase (Bancroft et al. 1988; Edler et al. 2007). There have been reports on a fat preference in rats in relation to the allopregnanolone precursor progesterone. In this study, allopregnanolone‐treated rats increased the intake of the food with the highest fat content, cookies which contain 25% fat, compared to chow which contains less than 5% fat. Fat intake of female rats peaks during the phase of the estrus cycle when progesterone levels are elevated (Leibowitz et al. 1998). Allopregnanolone levels during the estrus cycle correlate with the elevated progesterone levels (Ichikawa et al. 1974; Genazzani et al. 1998). Also, injections of progesterone to ovariectomized estrogen‐treated rats resulted in a significant increase in fat intake, while it had no effect on intake of carbohydrates and protein (Leibowitz et al. 2007). Human data on energy intake during the menstrual cycle are more diverse. Premenstrual increased intake of both fats and carbohydrates have been reported, but there are several reports which discusses increases in fat intake primarily (Tarasuk and Beaton 1991; Johnson et al. 1994; Martini et al. 1994). Gong et al. 1989 investigated possible influence of menstrual cycle phases on sucrose intake, but found no significant correlations (Gong et al. 1989). The elevated intake of the more fat containing cookies in this study together with results of progesterone effects on food selection might suggest that allopregnanolone could have an impact on macronutrient selection favoring fat intake. However, more research to evaluate this hypothesis is required.

It has not been clarified if the allopregnanolone‐induced hyperphagia is more related to reward aspects or more related to hunger aspects of feeding. The experimental setup in this study was based on food preference protocols used previously to investigate the effect of opioids and ghrelin, that is, two different food items were simultaneously presented in a choice situation (Giraudo et al. 1999; Bomberg et al. 2007). The results showed that allopregnanolone in a choice situation increased the consumption of cookies and not chow, that is, the energy‐rich and palatable food, while when the choice was between chow and sucrose only the intake of chow and not that of the palatable sucrose was increased. This would indicate that allopregnanolone increases the drive for more energy‐rich food over the more palatable food. It thus seems as allopregnanolone makes the rats more prone to eat the food that have higher energy density even though we cannot rule out some influence of palatability.

## Conclusions

In summary, we report that allopregnanolone preferentially increased the consumption of the more calorie dense food offered in a choice situation. This was seen both in the choice between a 10% sucrose solution and chow as well in the choice between Maryland Original^™^ chocolate chip cookies and chow. Due to this it could be speculated that allopregnanolone favor a choice of diet that is associated with obesity.

## Acknowledgments

The authors thank Magnus Löfgren for valuable suggestions during the planning of the experiments. The authors also thank Per Lundgren, Samira Gouissem, Martina Johansson and Karin Wallgren for their excellent assistance in the animal experiments.

## Competing interests

Torbjörn Bäckström is shareholder of Umecrine AB.
